# The So-Called Riggs' Disease and Its Treatment

**Published:** 1878-04

**Authors:** George A. Mills

**Affiliations:** Brooklyn.


### ARTICLE IV.

### *Directions as to the Treatment of the So-called Riggs' Disease, with the Instruments Devised by Dr. Riggs.*

BY GEORGE A. MILLS, BROOKLYN.

Dr. Riggs' instruments are six in number. Their peculiar value is in their forms, which make it possible to reach the remotest parts of the diseased structures. Nos. 1 and 2 are rights and lefts, and are designed to be used on the approximal and buccal surfaces of the molars, generally from, instead of towards, the operator. Nos. 3 and 4 are also rights and lefts, with the cutting edge towards the handle, and are intended to be used on all the surfaces of the ten anterior, superior, and inferior teeth, and also on the lingual and palatal surfaces of the molars. No. 5 has two cutting edges towards the handle, is thin, and spring-tempered; to be used between teeth closely approximating to each other. No. 6 has two cutting edges towards the handle, and corresponds in shape to Nos. 3 and 4, and is for general use.

In order to facilitate the operation, and to be sure that the ground has been thoroughly gone over, it is safer to establish something of a system in the operation. For instance, if the anterior inferior teeth are to be operated upon, commence at the mesial line, with instrument No. 3; standing on the right side of the patient, with the left arm thrown around the patient's head to the left side; the fingers of the hand placed under the chin, and the thumb resting on the cutting edge of the teeth or on the lip; the right hand grasping the instrument, with the thumb resting upon the teeth or jaw for support, and beginning at the mesial line on the proximate surface of the right central, drawing the scaler with a circular motion towards the labial. Having completed the operation on that part of the tooth, not only to the extreme point of the disease, but beyond and into the live tissue (the operator can detect by the feeling, which is transmitted readily to a hand trained to a delicate sense of touch, the difference between living and necrosed tissue,) pass to the next tooth, cleansing the same surfaces in the same manner to the same point, and continuing on each tooth to the posterior approximal surface of the second bicuspid, as a general rule. From this point, taking up instrument No. 1, pass on with it to the third molar in the same manner, with the exception that the movement should be from, instead of towards, the operator. Then coming back to the mesial line, with instrument No. 4 operate on the same surfaces in like manner to the same point on the left side, up to the point at which instrument No. 1 was taken up. Then substituting instrument No. 2, pass on to the third molar, as before directed. We have now been operating from right to left on both approximal and labial surfaces. We now consider the operation about half completed. We again come back to the mesial line and operate from left to right in the same manner; this being done, we have completed the operation on the lower teeth, with the exception of the lingual surfaces. We again commence at the mesial line, and operate from right to left, and from left to right, using instruments Nos. 3, 4, and 6, according to convenience. This being done, the operation on the lower teeth is supposed to be completed so far as the Riggs instruments are concerned.

Experience, which is the best teacher, will direct the operator in all the different stages of the disease that may come under his notice. It will require no little patience, perseverance, and experience to become an expert in this operation, but the reward will come through success, and will be a great compensation. No operation performed by the dentist in the mouth is more appreciated by patients after they have seen the results. It is really a surgical operation, and is of vastly more importance than has been attached to it by the profession generally. The teeth and all the exposed portion of the roots must now be polished; the nicer the better. A very efficient method of polishing the necks and crowns, and even down on the roots in many instances, is by the use of various widths of *tape*, with pumice or other polishing powders. To make the tape efficient in covering the entire surface, double it in the form of the letter U, placing it over the tooth to be polished, and taking the ends and crossing the tape around the tooth, giving a backward and forward motion, quickly and gently, up and down. A clean, polished surface can thus be secured more quickly and more perfectly than in any other way. A soft string used in the same way is efficient, as are also all the polishing appliances used in connection with the dental engine. After this operation is completed, have the patient rinse the mouth with lukewarm salt-water; the salt in the proportion of a teaspoonful to a goblet of water. Now pack around the teeth a sufficient supply of Japanese paper, and absorb the excess of moisture, then apply the officinal aromatic sulphuric acid with the Farrar syringe, which is doubtless the neatest and most efficient instrument we have at the present time, because of the long and delicately-constructed gold tubes, and the method of forcing the acid direct to the parts, and avoiding its escape into the mouth, by means of the screw on the piston. This completes the entire operation upon the lower teeth. The patient should be directed to wash the mouth thoroughly from day to day with lukewarm salt-water; and if inconvenienced by tenderness or pain about the teeth, may be instructed to apply tincture of myrrh, which will afford immediate comfort. Give earnest and intelligent directions as to the manner in which the teeth should be brushed; not crossways, but with a rotating motion, the inferior teeth from the gums up, and the superior teeth the reverse. Impress upon the patient the importance of more than ordinary effort, and give the assurance that this method of brushing, faithfully carried out, will prevent the return of the deposit upon the teeth. This I give as an answer, which is incontrovertible, to the remarks so often made, that if the deposits are removed they will again return. I say it without hesitation, for I have been making observations of late that prove it to be true, that faithful brushing by patients, under the instruction of the operator, will prevent it. Should the case which has been treated have advanced towards the third or fourth stages, as described in former articles, the amount of morbid tissue which might be more or less present can be brought more expeditiously to a healthy action by local treatment with tincture of iodine, but it will generally be found that the use of aromatic sulphuric acid will be sufficient. In extreme cases the patient should be required to revisit the operator, as directed by him, that he may watch the progress of the case and apply the remedies that may be indicated. At the expiration of about four weeks the congested tissue will have been displaced by a more healthy one. The case will now admit of a *revision* at such points as may indicate the necessity. In extreme cases, I have noticed a general improvement going on over a period of time extending to six months; giving indications at that time of an amount of success that the second or third month did not show.

I have perhaps said enough in these articles to convince not a few of the importance of this matter. Many have spoken to me regarding it, and have asked how great a percentage of the cases are amenable to treatment. Dr. Riggs claims *ninety per cent.* I think it true *in his hands;* in my own, the percentage increases with my experience.—*Dental Cosmos.*

